# Crosstalk between integrin αvβ3 and ERα contributes to thyroid hormone-induced proliferation of ovarian cancer cells

**DOI:** 10.18632/oncotarget.10757

**Published:** 2016-07-21

**Authors:** Meng-Ti Hsieh, Le-Ming Wang, Chun A. Changou, Yu-Tang Chin, Yu-Chen S.H Yang, Hsuan-Yu Lai, Sheng-Yang Lee, Yung-Ning Yang, Jacqueline Whang-Peng, Leroy F. Liu, Hung-Yun Lin, Shaker A. Mousa, Paul J. Davis

**Affiliations:** ^1^ Taipei Cancer Center, Taipei Medical University, Taipei, Taiwan; ^2^ The PhD Program for Cancer Biology and Drug Discovery, College of Medical Science and Technology, Taipei Medical University, Taipei, Taiwan; ^3^ Department of Obstetrics and Gynecology, Wan Fang Hospital, Taipei Medical University, Taipei, Taiwan; ^4^ Integrated Laboratory, Center of Translational Medicine, Taipei Medical University, Taipei, Taiwan; ^5^ Core Facility, Taipei Medical University, Taipei, Taiwan; ^6^ Department of Dentistry, Wan-Fang Medical Center, Taipei Medical University, Taipei, Taiwan; ^7^ School of Dentistry, Taipei Medical University, Taipei, Taiwan; ^8^ Center for Teeth Bank and Dental Stem Cell Technology, Taipei Medical University, Taipei, Taiwan; ^9^ Joint Biobank, Office of Human Research, Taipei Medical University, Taipei, Taiwan; ^10^ Department of Pediatrics, E-DA Hospital, I-Shou University, Kaohsiung, Taiwan; ^11^ Pharmaceutical Research Institute, Albany College of Pharmacy and Health Sciences, Albany, New York, USA; ^12^ Department of Medicine, Albany Medical College, Albany, New York, USA

**Keywords:** thyroid hormone, integrin αvβ3, ERα crosstalk, ovarian cancer

## Abstract

Ovarian cancer is the leading cause of death in gynecological diseases. Thyroid hormone promotes proliferation of ovarian cancer cells *via* cell surface receptor integrin αvβ3 that activates extracellular regulated kinase (ERK1/2). However, the mechanisms are still not fully understood. Thyroxine (T_4_) at a physiologic total hormone concentration (10^−7^ M) significantly increased proliferating cell nuclear antigen (PCNA) abundance in these cell lines, as did 3, 5, 3′-triiodo-L-thyronine (T_3_) at a supraphysiologic concentration. Thyroid hormone (T_4_ and T_3_) treatment of human ovarian cancer cells resulted in enhanced activation of the Ras/MAPK(ERK1/2) signal transduction pathway. An MEK inhibitor (PD98059) blocked hormone-induced cell proliferation but not ER phosphorylation. Knock-down of either integrin αv or β3 by RNAi blocked thyroid hormone-induced phosphorylation of ERK1/2. We also found that thyroid hormone causes elevated phosphorylation and nuclear enrichment of estrogen receptor α (ERα). Confocal microscopy indicated that both T4 and estradiol (E_2_) caused nuclear translocation of integrin αv and phosphorylation of ERα. The specific ERα antagonist (ICI 182,780; fulvestrant) blocked T_4_-induced ERK1/2 activation, ERα phosphorylation, PCNA expression and proliferation. The nuclear co-localization of integrin αv and phosphorylated ERα was inhibited by ICI. ICI time-course studies indicated that mechanisms involved in T_4_- and E_2_-induced nuclear co-localization of phosphorylated ERα and integrin αv are dissimilar. Chromatin immunoprecipitation results showed that T_4_-induced binding of integrin αv monomer to ERα promoter and this was reduced by ICI. In summary, thyroid hormone stimulates proliferation of ovarian cancer cells via crosstalk between integrin αv and ERα, mimicking functions of E_2_.

## INTRODUCTION

Thyroid hormones (L-thyroxine, T_4_; 3, 5, 3′-triiodo-L-thyronine, T_3_) are a proliferation factor *in vitro* for a variety of cancer cells [1–8]. They stimulate cell proliferation via a cell-surface receptor on integrin αvβ3 [1]. This receptor is at or near the arginine–glycine–aspartate (RGD) recognition site on the integrin that is involved in the interaction of the integrin with extracellular matrix proteins [9, 10]. Downstream of integrin are the signal transduction molecules that may be extracellular-regulated kinases 1 and 2 (ERK1/2) [1], and we have shown that T_4_ rapidly increases cellular ERK1/2 activity via the integrin [11, 12] or exclusively for T_3_, PI3-kinase via Src kinase to stimulate TRβ trafficking.

Nuclear TRβ does not play a primary role in the thyroid hormone via integrin αvβ3-initiated actions [9, 13]. However, overexpression of TRβ1 can be involved in thyroid hormone (T_3_)-induced inhibition of proliferation of certain cells [14]. We have also shown that thyroid hormone can act at the cell surface on the integrin receptor and influence expression of hypoxia-inducible factor-1α (HIF-1α), which is PI3-kinase-dependent [12].

Ovarian cancer develops when a mutation or genetic change occurs in the cells on the surface of the ovaries or in the fallopian tubes and leads to uncontrolled cell growth that may often metastasize [15]. Ovarian cancer is also a thyroid hormone-dependent neoplasm [9]. T_3_ has been shown to directly exert inflammatory effects on ovarian surface epithelial cell function *in vitro* and activate expression of genes associated with inflammation, including *COX2*, *MMP9*, and *HSD11B1* [8, 16]. Studies also indicate that T_3_ increases the expression of *ERα*, which strongly associates with the development of epithelial ovarian cancer, which may explain the epidemiological linkage between hyperthyroidism and ovarian cancer [16].

The proliferative effect of thyroid hormone on the induction of ERK1/2-dependent serine phosphorylation of estrogen receptor α (ERα, S167) mimics the effect of estrogen in ERα-positive breast cancer [5] and non-small cell lung cancer cells [3]. This effect of thyroid hormone can be blocked by the ER antagonist, ICI 182,780. Thus, there is a crosstalk between thyroid hormone and estrogen signaling pathways in certain cancer cells; these pathways originate non-genomically outside the nucleus and require ERK1/ERK2, but culminate in specific intranuclear events.

In the experiments described here, thyroid hormone is shown to induce the proliferation of human ovarian cancer cells via crosstalk between integrin αvβ3 and ERα. ICI 182,780 inhibited integrin αv binding with *ERα* promoter in the ChIP assay and inhibited ERK1/ERK2 activation and cell proliferation in *ERα* bearing ovarian cancer cells. These results indicate that thyroxine induced cell proliferation occurs via crosstalk between integrin αvβ3 and ICI 182,780 (fulvestrant)-sensitive signal transduction pathways. These findings also suggest a mechanism whereby thyroid hormone status might enhance the proliferation and estrogenic sensitivity of the ovarian cancer cells and thereby accelerate both the progress and the treatment of ovarian cancer.

## RESULTS

### Thyroid hormone activates ERK1/2 and proliferation in ovarian cancer cells

**Figure 1:**
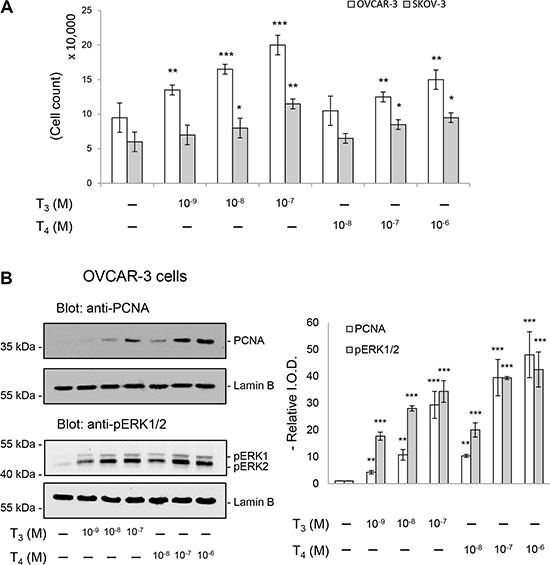
Thyroid hormone induced proliferation in ovarian cancer cells OVCAR-3 and SKOV-3 were treated with T_3_ (10^−9^ to 10^−7^ M) or T_4_ (10^−8^ to 10^−6^ M) for 3 days (**A**), 24 hours (B, upper panel) or 30 min (B, lower panel). Three independent sets of cells were harvested at indicated time for later analysis. (A) Cells were harvested and counted directly. Compared to control: *p* < 0.05 :*p < 0.01 :** (**B**) OVCAR-3 cells pellets were resolved by SDS-PAGE. PCNA and phosphorylated ERK1/2 antibodies were used for Western blotting. Quantitative results were plotted as bar chart with SD. Compared to control: *p* < 0.01: **p < 0.001:***

Thyroid hormone-induced cell proliferation was examined by cell count and MTT assay (Figure 1A). When ovarian cancer OVCAR-3 and SKOV-3 cells were treated with L-thyroxine (T_4_) (10^−8^ to 10^−6^ M) daily for 3 days with refreshed medium with T_4_, cell proliferation increased with dosage effect (Figure 1A). Similar results were obtained with 3,5,3′-triiodo-L-thyronine (T_3_) (10^−9^ to 10^−7^ M) (Figure 1A) In order to examine the effect of thyroid hormone on signal transduction and cell proliferation in ovarian cancer cells, OVCAR-3 cells were treated with different concentrations of thyroid hormones (T_3_ or T_4_) for 30 min. Both T_3_ and T_4_ induced activation of MAPK (ERK1/2) with 30 min treatment (Figure 1B). Parallel studies were conducted to treat cells with thyroid hormone for 24 h. The accumulation of proliferating cell nuclear antigen (PCNA) increased in T_4_- and T_3_-treated cells (Figure 1B).

### Integrin αvβ3 is involved in thyroid hormone-induced signaling and proliferation in ovarian cancer cells

**Figure 2:**
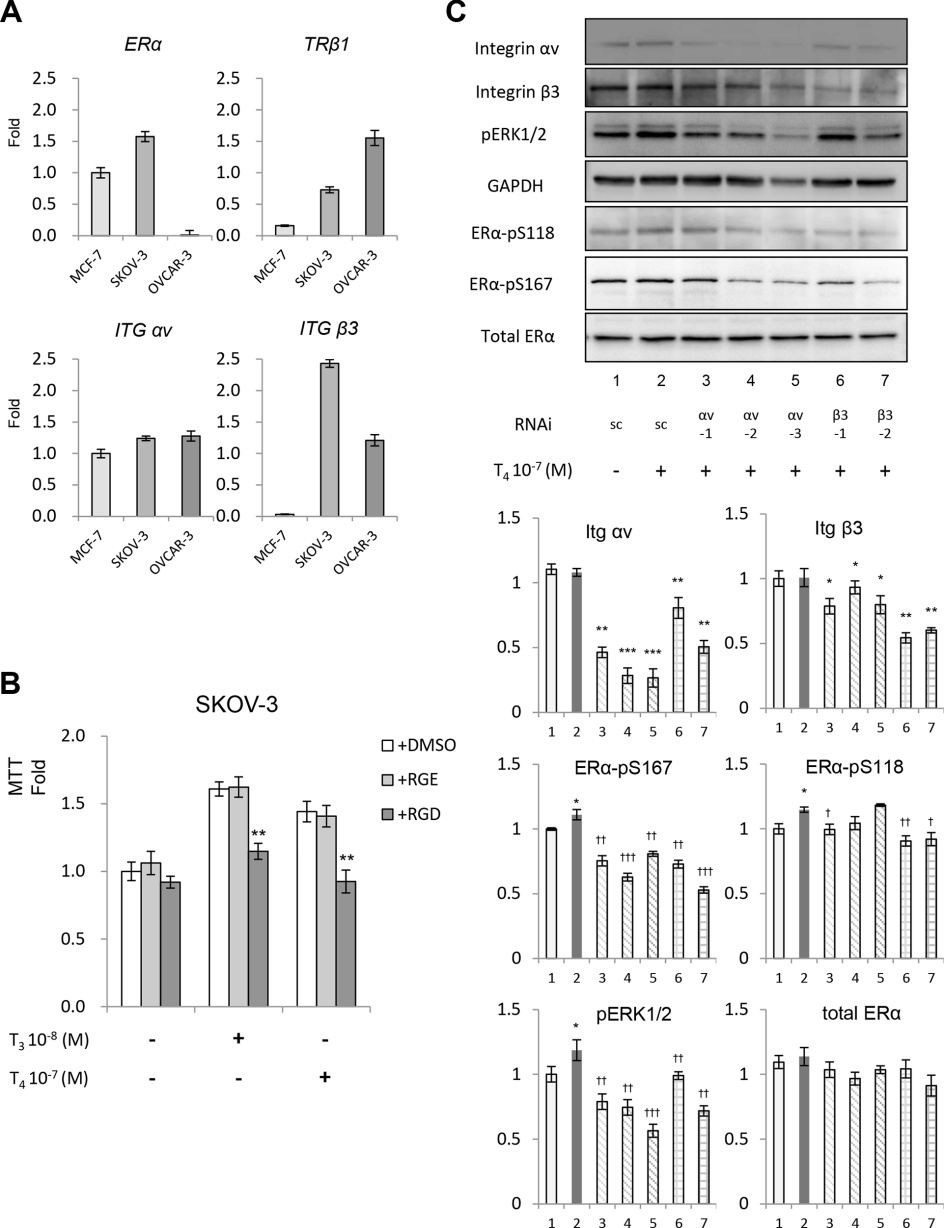
Thyroid hormone induced phosphorylation of ERα (**A**) mRNA levels of *ERα*, *TRβ1*, *ITG αv* and *ITG β3* from MCF-7, SKOV-3 and OVCAR-3 were measured by quantitative real-time PCR and normalized with 18Sr RNA. Results were expressed as folds of increase. (**B**) SKOV-3 cells were treated in the presence or absence of indicated peptides with T_3_ or T_4_ for 3 days, cells in 96 wells were subjected to the MTT assay. (**C**) scramble or shRNA of integrin αv or β3 were transiently transfected to SKOV-3 cells and treated with thyroid hormone (T_4_) for 30 min, and whole cell lysates were harvested for western blot analysis. Indicated antibodies were used. Compared to sc: *p* < 0.05 :*p < 0.01 :**p < 0.001 :*** ; Compared to sc treated with T_4_: *p* < 0.05: ^†^*p* < 0.01: ^††^*p* < 0.001: ^†††^

Thyroxine has been shown to induce cell proliferation via activating ERα in breast cancer MCF-7 cells [5] and non-small cell lung cancer NCI-H522 cells [3]. The estrogen receptor, ERα, is variably expressed in ovarian cancer cells, as shown in Figure 2A. In SKOV-3, estrogen receptor α (*ERα*), thyroid hormone receptor β1 (*TRβ1*) and integrin β3 (*ITG β3*) are higher than in breast cancer cell line MCF-7. On the other hand, OVCAR-3 cells had higher *TRβ* but lower *ITG β3*, while *ERα* was barely detected (Figure 2A). Because SKOV-3 cells contain both integrin αvβ3 and ERα, we considered it a suitable model for studying for the possible existence of crosstalk between these two proteins. These cells were used in the later experiments. The involvement of integrin αvβ3 in thyroid hormone-induced proliferation was demonstrated by Arg-Gly-Asp (RGD) peptide and control RGE peptide (Figure 2B). The RGD recognition site on the integrin is at or near the thyroid hormone receptor site [18, 20, 21]. In anaplastic ovarian cancer cells pre-incubated for 30 min prior to treatment with 10^−7^ M T_4_ or 10^−8^ M T_3_, RGD peptide (50 nM), but not RGE peptide (50 nM), inhibited thyroid hormone action on cell proliferation. These results suggest that thyroid hormone acts via integrin αvβ3 to induce ERK1/2 activation and proliferation.

### Thyroid hormone activates ERα in ovarian cancer cells

To demonstrate the thyroid hormone binding site on the ovarian cancer cell surface integrin αvβ3 that plays a role in hormone-induced ERK1/2 activation, experiments were conducted involving *shRNA* of αv or β3 to reduce the expression of integrin αv or β3, and three αv and two β3 clones were selected for the assay. Knocked down effects of integrin levels were shown by western blot. The reduction of integrin expression also inhibited thyroxine-induced ERK1/2 activation (Figure 2C), implicating integrin αvβ3 is involved in the activation of MAPK by thyroid hormone in ovarian cancer cells. Knock down of both integrins also reduced phosphorylation of ERα, indicating the possibility of signal transduction from integrin to ERα.

**Figure 3:**
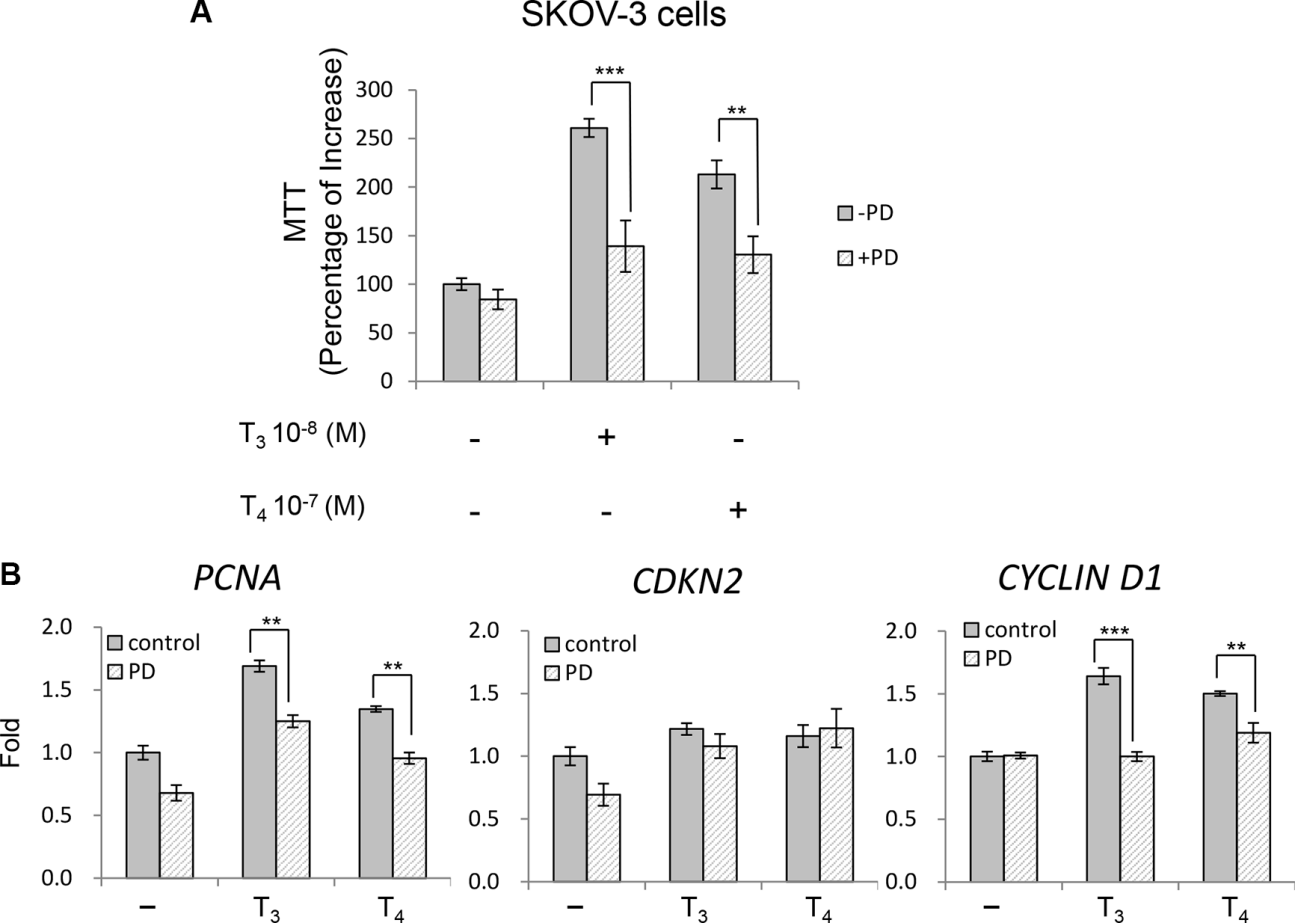
PD 98059 suppressed thyroid hormone-induced proliferation SKOV-3 cells were treated in the presence or absence of PD (30 μM) with T_3_ (10^−8^ M) or T_4_ (10^−7^ M) for 3 days (**A**) or 24 hours (**B**). Cells in 96 wells were subjected to the MTT assay (A) and mRNA levels of proliferation markers were quantified from cell pellets by qPCR (B). *p* < 0.01:**p < 0.001 :***

An inhibitor of the MAPK signal transduction pathway at MEK, PD 98059 (PD) (30 μM) inhibited the proliferative effect of thyroid hormone T_3_ and T_4_ on ovarian cancer cells SKOV-3 (Figure 3A). When SKOV- 3 cells were treated with PD in the presence of either T_3_ or T_4_ and for three days, both T_3_ and T_4_-induced cell proliferation was inhibited. The same setup of cells was collected after 24 hours treatment, and the expression levels of proliferation markers *PCNA*, *CDKN2* and *CYCLIN D1* were measured by quantitative real-time PCR (Figure 3B). Both *PCNA* and *CYCLIN D1* were reduced under PD exposure while *CDKN2* did not change significantly. These results link the activation of ERK1/2 by thyroid hormone to cancer cell proliferation.

### Thyroid hormone stimulates cell proliferation via cross-talk between integrin αv and ERα in ovarian cancer cells

**Figure 4:**
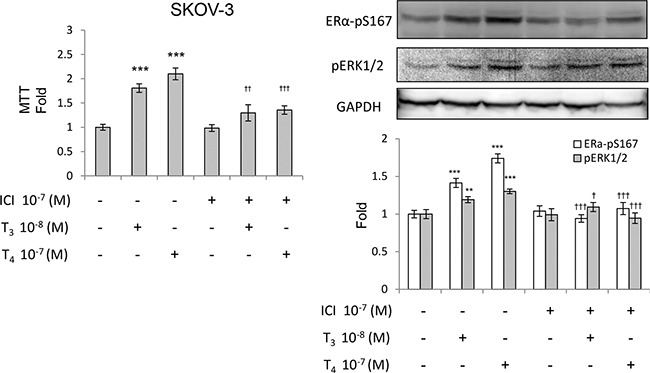
Thyroid hormone-induced phosphorylation of ERα was suppressed by ICI SKOV-3 cells were pre-treated with ICI for 30 min prior to an additional 30 min of indicated thyroid hormone treatment and kept for 72 h (MTT assay) or collected immediately after treatment. Total cell lysates were harvested for western blot analyses. Antibodies used were as indicated. The quantitative data were normalized by GAPDH and are displayed as a bar chart. Compared to control: *p* < 0.01:**p < 0.001:***; compared to treated with indicated thyroid hormone alone: *p* < 0.05: ^†^*p* < 0.01: ^††^*p* < 0.001: ^†††^

Thyroxine induces integrin αv translocation into nuclei and association with p300 [22]. It suggested that nuclear integrin αv may play a role in thyroid hormone-dependent gene transcription. In order to examine the relationship between integrin αv and ERα on gene regulation, SKOV- 3 cells were treated with T_4_ and T_3_ in the presence or absence of a specific inhibitor of ERα, ICI (Figure 4). ICI inhibited thyroid hormone-induced proliferation. Both phosphorylation of ERα (S167) and ERK1/2 induced by thyroid hormone were suppressed by ICI.

**Figure 5:**
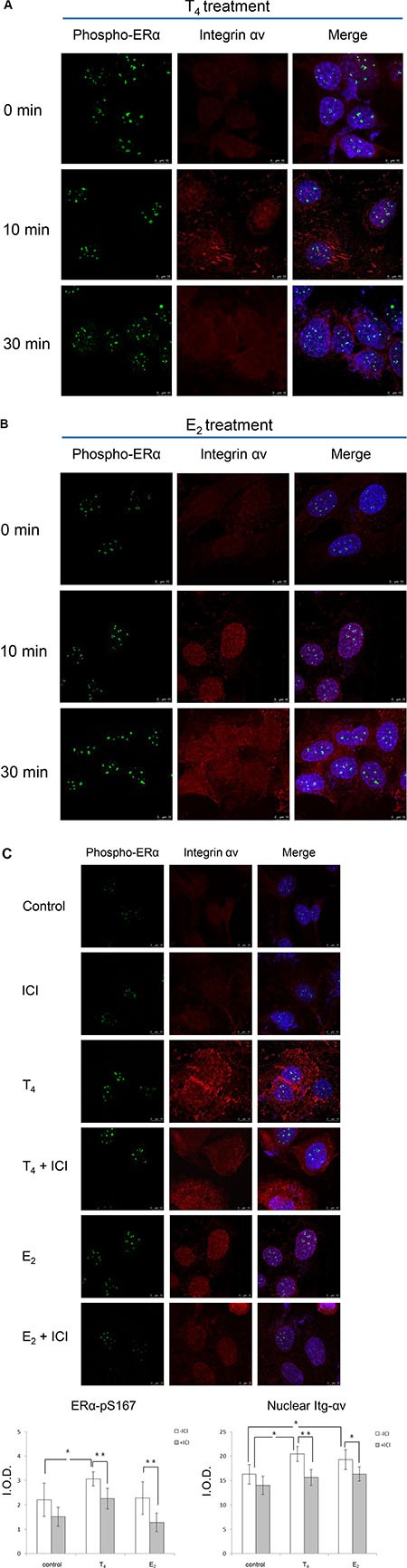
Thyroid hormone and estrogen induce phosphorylation of ERα and nuclear integrin αv translocation SKOV- 3 cells were treated with (**A**) thyroid hormone, (**B**) estrogen, for different periods of time as indicated. (**C**) SKOV-3 cells treated with thyroid hormone or estrogen for 10 min in the absence or presence of ICI were fixed and stained with anti-integrin αv and phospho-ERα (S167) antibodies, and subsequently with fluorescent secondary antibodies. Nuclear punctate of phosphorylated ERα-induced by T_4_ (shown in green) were increased with time and co-localized with integrin αvβ3 (shown in red) to yield a yellow color. Nuclei were stained with DAPI and showed as blue. The phosphorylation of ERα and nuclear translocation of integrin αv were inhibited by ICI. Quantitative fluorescence intensities are shown as average intensity per cell. *p* < 0.05: *p < 0.01: **

Confocal microscopy (Figure 5A) showed that thyroid hormone caused time- dependent nuclear translocation of phosphorylated ERα in ovarian cancer SKOV-3 cells. The nuclear accumulation of phosphorylated ERα induced by T_4_ (shown in green) co- localized with integrin αv (shown in red) to yield a yellow color. Similar results were observed in E_2_- treated SKOV-3 cells (Figure 5B). However, the nuclear accumulation of integrin αv and phosphor-ERα appeared to occur at a slower rate than with T_4_. The action of T_4_ was inhibited by co-incubation of T_4_ and ICI (Figure 5C). Interestingly, T_4_-induced nuclear accumulation of integrin αv appeared to be less sensitive to ICI than estrogen-directed nuclear translocation of av monomer. These results suggest that T_4_-induced nuclear accumulation of integrin αv may include an additional step upstream of ICI-ERα interaction. ICI has previously been shown to block thyroid hormone-stimulated activities in human breast cancer MCF-7 cells [5] and non-small cell lung cancer NCI-H522 cells [3]. We confirmed here that ICI blocked E_2_-induced ERα phosphorylation and then showed that ICI prevented nuclear co-localization of integrin αv and phospho-ERα (Figure 5C). At 10 min, ICI blocked 26.5% and 23.5% of T_4_-induced nuclear ERα phosphorylation and nuclear integrin αv accumulation, respectively, indicating a simultaneous effect of thyroid hormone on integrin and ERα. On the other hand, ICI blocked 43.7% and 15% of E_2_-induced nuclear ERα phosphorylation and nuclear integrin αv accumulation, respectively, thus suggesting a sequential effect of estrogen on integrin and ERα.

**Figure 6:**
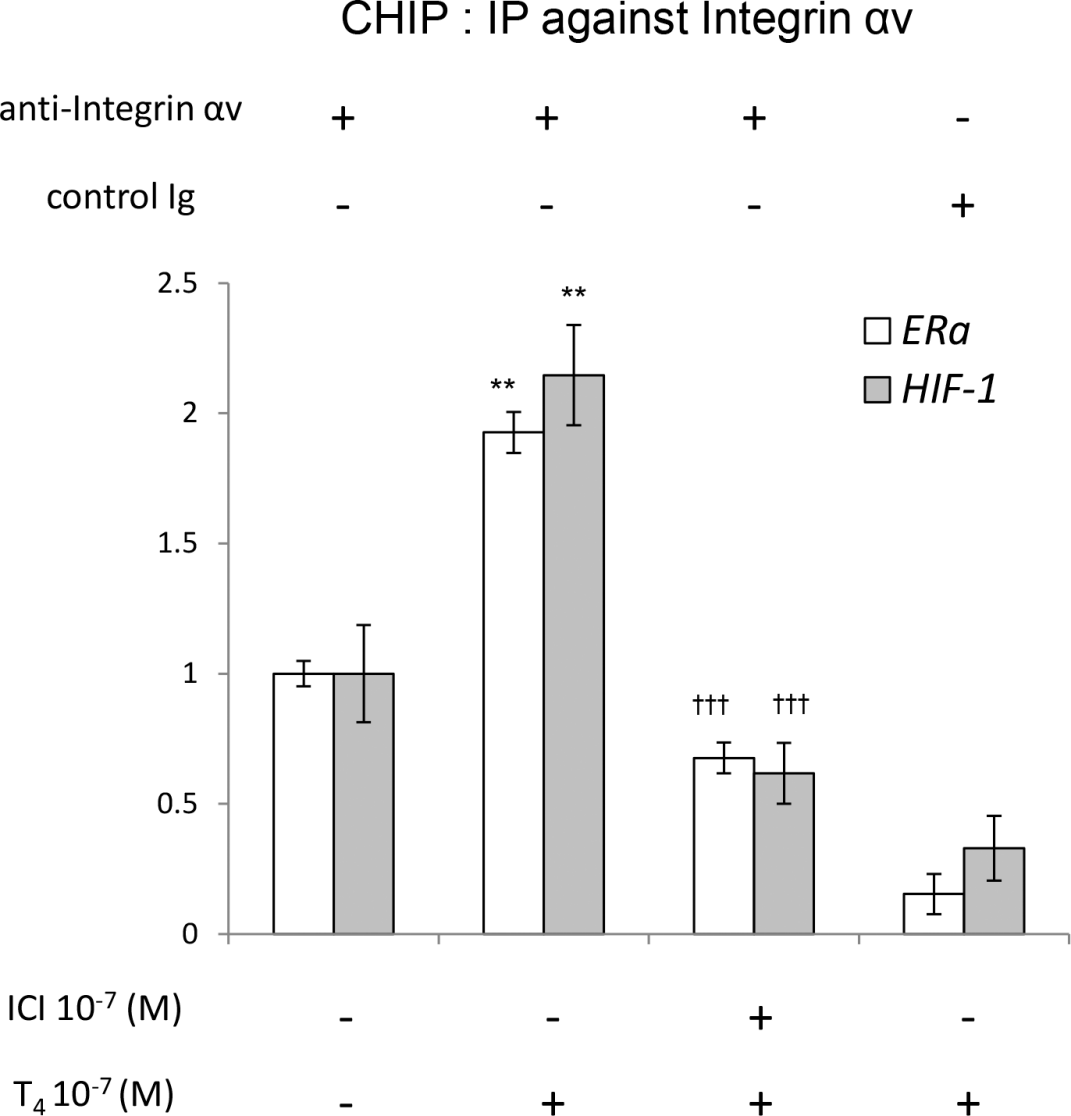
Crosstalk between integrin αvβ3 and ERα SKOV-3 were pre-treated in the presence or absence of ICI for 30 min prior to another 30 min of T_4_ treatment and harvested for ChIP. Total cell lysate was immunoprecipitated with anti-integrin αv and pulled-down DNA was measured with qPCR. Compared to control: *p* < 0.01: **; Compared to T_4_ alone: *p* < 0.001: ^†††^

We further studied the role of ERα on thyroid hormone-induced proliferation in ovarian cancer cells. A chromatin immunoprecipitation assay (ChIP) was conducted by using anti-integrin αv antibody. Mouse IgG was used as a negative control. T_4_ increased integrin αv binding to the *ERα* promoter except the one with mouse IgG (Figure 6, upper panel). The T_4_-activated integrin αv formed a complex with the *ERα* promoter as well as with *HIF-1* promoter, which was reduced by ICI (Figure 6, lower panel). The binding of integrin αv to the ERα promoter sequence is specific. These results suggest that crosstalk between integrin αvβ3 and ERα is involved in T_4_-dependent transcription.

## DISCUSSION

Proliferation of ovarian cancer has been shown to be hormone-dependent. The association between sex hormones and gynecological cancers, including ovarian carcinoma, has been extensively described [23–25]. We have previously shown that thyroid hormone stimulates cell proliferation in breast cancers, gliomas and lung cancers. Rasool *et al*. measured thyroid hormone levels in breast cancer and ovarian cancer patients and found significant increases in both T_3_ (*P* = 0.000*) and T_4_ (0.005*) levels in breast cancer patients compared to healthy controls. For ovarian cancer patients, a significant increase was found only for T_4_ (*P* = 0.050) [15]. Extensive *in vitro* studies reviewed elsewhere have suggested that T_4_ may play a more important role than T_3_ in stimulating cancer cell proliferation [26].

The stimulatory effect of thyroid hormone on cancer cell proliferation is largely expressed via a cell surface receptor for the hormone on the extracellular domain of integrin αvβ3 [26], rather than via nuclear thyroid hormone receptors (see below). We showed in the current studies that thyroid hormones stimulated proliferation of ovarian cancer cells with high levels of integrin αvβ3 through phosphorylation of ERK1/2 (Figure 2). The growth promoting effects of thyroid hormone initiated at integrin αvβ3 were blocked by an inhibitory integrin ligand, RGD peptide, or by shRNA knockdown of either integrin αv or β3 monomer. We confirmed that thyroid hormone activation of ERK1/2 can be reduced by the MEK inhibitor PD 98059 (Figure 3). There are two thyroid hormone-binding domains on integrin αvβ3; the S1 domain is exclusively for T_3_ and S2 binds T_4_ and has a lower affinity for T_3_. After binding to S2, thyroid hormone activates the ERK1/2 signal transduction pathway [12], facilitates tumor cell proliferation and inhibits apoptosis.

Integrin αvβ3 expression varies among ovarian cancer cell lines [27, 28] and among primary cultures of cells from ovarian cancer patients [29, 30]. The degree of integrin αvβ3 expression has been shown to link with disease prognosis [27, 31]. In addition, there is overexpression of αvβ3 in blood vessels supporting ovarian carcinomas [29], suggesting integrin αvβ3 contributes to angiogenesis and metastasis of ovarian cancer. The αvβ3 in ovarian cancer has been emphasized as a therapeutic target in mouse models; inhibition of αvβ3 expression improves survival [28, 32–35] and the response to therapy is correlated with αvβ3 expression level [28].

In addition to the contribution from integrin αvβ3, ERα was also shown in the current studies to play a crucial role as an alternative signaling relay in thyroid hormone-induced proliferation in ovarian cancer cells (Figure 2C). Treatment of cells with thyroid hormone induced phosphorylation of nuclear ERα and the estrogen receptor inhibitor, ICI 182,780, blocked this activation (Figure 4), as did inhibition of αvβ3. These results indicated that ERα and αvβ3 can co-operate in ovarian cancer cells to enhance the proliferative effect of thyroid hormone. Confocal microscopy and biochemical data showed that integrin αvβ3 and ERα formed complexes apparently linked to ovarian cancer proliferation. Interruption of the complex formation led to partial inhibition of proliferation. These data may contribute to the hormone sensitivity differences observed between OVCAR-3 and SKOV-3; the former does not have detectable ERα, but does have integrin αvβ3, whereas the latter expresses both αvβ3 and a high level of ERα.

Nuclear thyroid hormone receptors TRα1, TRα2, and TRβ1 are present in primary ovarian surface epithelial cell cultures and it has been shown that one of these receptors (*TRβ1*) may, when mutated, be involved in pathologic actions of thyroid hormone [36]. Other studies indicate that T_3_ can stimulate the expression of *ERα* without affecting *ERβ1* or *ERβ2*. T_3_ also increases the expression of inflammation-associated genes such as cyclooxygenase-2, matrix metalloproteinase-9, and 11β hydroxysteroid dehydrogenase type 1 [16] that may contribute to cancer behavior.

In summary, we show here that the proliferative effects of thyroid hormone on ovarian cancer cells are initiated at integrin αvβ3 and may involve consequent crosstalk with ERα. Activation of ERK1/2 by thyroid hormone was integrin αvβ3-dependent and activated ERK1/2 was responsible for phosphorylation of ERα. On the other hand, estrogen may affect integrin αvβ3 and cytoplasmic ERα simultaneously to stimulate ERα and integrin αv translocation to the cell nucleus. Both signal pathways collaborate to promote ovarian cancer cell proliferation. These findings offer the possibility of new directions for ERα-positive ovarian cancer management that recognize the contributions of thyroid hormone in the absence of host estrogen.

## MATERIALS AND METHODS

### Cell lines

Human ovarian carcinoma (OVCAR-3 and SKOV- 3) cells were obtained from American Type Culture Collection (Manassas, VA, USA). Cells were maintained in RPMI medium supplemented with 20% FBS and 0.01 mg/ml bovine insulin (OVCAR-3) and 10% FBS (SKOV-3) and under 5% CO_2_/95% air at 37°C. Cells used in this study were kept within 15 passages. Prior to hormonal treatment, cells were placed in 0.25% hormone-stripped FBS-containing medium for 2 d. T_3_ or T_4_ (Sigma-Aldrich, USA) was then added to medium to achieve total hormone concentrations of 10^−7^ −10^−9^ (T_3_) or 10^−6^ −10^−8^ M (T_4_) that was directly measured in aliquots of representative media from hormone-treated cells [17, 18]. A total concentration of T_4_ of 10^−7^ M in the medium used yields a physiological free hormone level (18); total T_3_ concentrations in the range cited yield supraphysiologic free hormone concentrations. Hormone-containing medium was refreshed daily.

### MTT assay

Cells (2 × 10^3^ cells per well) were seeded in 96-well plates and untreated (controls) or treated (30 μM PD 98,059, (Selleck Chemicals, USA); 50 nM RGD or RGE peptide, (Sigma-Aldrich) for 72 h, and reagent-containing media were refreshed daily. Cell proliferation was determined by incubating the cells with 200 μL of fresh medium containing 1 mg/mL 3-(4,5-dimethylthiazol-2-yl)-2,5-diphenyltetrazolium bromide (MTT) (Sigma-Aldrich) for 4 h at 37°C. After removal of the MTT solution, the resulting formazan crystals were dissolved completely in an ethanol/dimethyl sulfoxide mixture (1:1) and the plates were read using a microplate reader (Anthos 2010; Biochrom, Cambridge, UK) by measuring the absorbance at 490 nm. Triplicate wells were assayed for each experiment and three independent experiments were performed. Data are expressed as the mean of OD490 ± SD.

### Transfection of siRNA

Ovarian cancer cells were seeded onto 6-well tissue culture plates at 60–80% confluence (10^5^cells /well), and maintained in the absence of antibiotic for 24 h before transfection. Just prior to transfection the culture medium was removed, and the cells washed once with PBS, then transfected with small interfering αv RNA or scrambled RNA (0.2 μg/well, RNAi core, Academia Sinica, Taipei) using Lipofectamine 2000 (2 μg/well) in Opti-MEM I medium according to the instructions from Ambion (Austin, TX, USA). After transfection, cultures were incubated at 37°C for 4 h and then placed in fresh culture medium. After an additional 24 h, the cells were subjected to study.

### Immunoblotting

OVCAR-3 and SKOV-3 cells were maintained in complete RPMI-1640 in the absence or presence of 30 μM PD 98,059 or 10^−7^ M ICI 182,780 (Selleck Chemicals). Nucleoproteins or whole cell lysates were prepared and separated on discontinuous SDS-PAGE, then transferred by electroblotting to PVDF membrane (Millipore, Bedford, MA, USA), as we have previously described [12, 19]. Membranes were blocked with 5% milk in Tris-buffered saline containing 0.1% Tween, and then incubated with selected antibodies overnight: PCNA (GTX100539, GeneTex Inc. CA, USA), Lamin B (GTX103292, GeneTex Inc.), integrin αv (SC9969, Santa Cruz Inc., Santa Cruz, CA, USA ), integrin β3 (SC6627, Santa Cruz), phospho-ERK1/2 (4377S, Cell Signaling Technology, Danvers, MA, USA), total-ERα (8644S, Cell Signaling Technology), ERα-pS118(2511p, Cell Signaling Technology), ERα-pS167 (5587p, Cell Signaling Technology). Horseradish peroxidase (HRP)-conjugated secondary antibodies were either goat anti-rabbit IgG or goat anti-mouse IgG (1:1000, Dako, Carpenteria, CA, USA), depending on the origin of the primary antibody. Immunoreactive proteins were detected by chemiluminescence.

### Confocal microscopy

SKOV-3 ovarian cancer cells were exponentially grown on sterilized cover glass (Paul Marienfeld GmbH & Co. KG, Lauda-Königshofen, Germany) and treated with 10^−7^ M ICI 182,780 for 30 min prior to the treatment of 10^−7^ M thyroxine or 10^−9^ M E_2_ for different time periods. The cells were immediately fixed with 4% paraformaldehyde in phosphate buffered saline (PBS) for 20 min and then permeabilized in 0.1% Triton X-100 in PBS for 20 min. The cells on the slides were incubated with integrin αv antibody (1:200, Santa Cruz) or anti- ERα-phospho(S167)- antibody (1:200, Cell Signaling Technology) overnight at 4°C. Then cells were incubated with Alexa Fluor^®^-488 and Alexa Fluor^®^-647-conjugated secondary antibody (Abcam, Cambridge, United Kingdom) and mounted in EverBrite Hardset mounting medium with DAPI (Biotium, CA, USA). The fluorescent signals from integrin αv and phospho-ERα were recorded and analyzed with TCS SP5 Confocal Spectral Microscope Imaging System (Leica Microsystems, Wetzlar, Germany). The figures shown are representative of four fields for each experimental condition. Nuclei were defined by DAPI staining and nuclear fluorescence intensities were measured by ImageJ freeware (ImageJ, NIH, USA); data are shown as average intensity per cell.

### Quantitative real-time PCR

Total RNA was extracted using the RNeasy Micro Kit (Qiagen, Venlo, Netherlands) and cDNA was synthesized with the qScript™ cDNA Supermix (Quanta Biosciences, Gaithersburg, MD). Quantitative PCR was conducted with 5 μL of DNA combined with 10 μL of Perfecta SYBR Green FastMix (Quanta Biosciences), 0.3 μL each of 20 μM forward and reverse primers, and 4.7 μL DNase/RNase free water in a MicroAmp™ Optical 384-Well Reaction Plate (Applied Biosystems). The sequences for the primers amplified are: Homo sapiens proliferating cell nuclear antigen (*PCNA*), forward 5′-TCTGAGGGCTTCGACACCTA-3′ and reverse 5′-TCA TTGCCGGCGCATTTTAG-3′ (Accession No.: BC062439.1); Cyclin D1 (*CCND1*), forward 5′-CAAGGCCTGAACC TGAGGAG-3′ and reverse 5′-GATGACTCTGGAGAGG AAGCG-3′ (Accession No.: NC_000011.10); cyclin-dependent kinase inhibitor 2A (*CDKN2*), forward 5′-ACTGCGCTGCAGGTTATGAA-3′ and reverse 5′-AGCG AAACCAGTTCGGTCTT-3′ (Accession No.: NC_000009.12); Estrogen receptor α (*ERα*), forward ‘5-TAACCTCGG-GC TGTGCTCTT-3′ and reverse 5′-TTCCCTTGGATCTGAT GCAGTAG-3′ (Accession No.: NC_000006.12); thyroid hormone receptor β (*TRβ1*), forward 5′-AATGTCTGA- AGCCTGCCTAC-3′ and reverse 5′-GGCTTTGTCACCA CACACTA-3′ (Accession No.: NC_000003.12); integrin αv (*ITG αv*), forward 5′-TCCGATTCCAAACTGGGAGC- 3′ and reverse 5′-AAGGCCACTGAAGATGGAGC-3′ (Accession No.: NC_000002.12); integrin β3 (*ITG β3*), forward 5′- CTGGTGTTTACCACTGATGCCAAG-3′ and reverse 5′- TGTTGAGGCAGGTGGCATTGAAGG-3′ (Accession No.: NM_000212.2); hypoxia-inducible factor 1 α (*HIF-1α*), forward 5′-CCATGAAGAGTTGAGAG AGATGCT-3′ and reverse 5′-CTC-TGTGTTTTGTTCCTT GGTCTTT-3′ (Accession No.: NC_000014.9); glyceraldehyde-3-phosphate dehydrogenase (*GAPDH*), forward 5′- TGCCAAATATGATGACATCAAGAA-3′ and reverse 5′- GGAGTGGGTGTCGCTGTTG-3′ (Accession No.: NM_002046). The real time PCR reactions were performed using QuantiNova^TM^ SYBR^®^ Green PCR Kit (Qiagen) on CFX Connect™ Real-Time PCR Detection System (Bio-Rad Laboratories, Inc., Hercules, CA, USA) using the following conditions: 2 min at 50°C, 10 min at 95°C, 40 cycles of 15 sec at 95°C, and 1 min at 60°C. Data calculations of relative gene expression (normalized to GAPDH reference gene) were performed according to the ΔΔCT method. Fidelity of the PCR reaction was determined with melting temperature analysis.

### Data analysis and statistics

Immunoblot and nucleotide densities were analyzed with IBM^®^ SPSS^®^ Statistics software (SPSS Inc., Chicago, IL, USA). Two tails student's *t*-test was conducted and considered significant at *p*-values < 0.05 (*, or ^†^), 0.01 (**, or ^††^), 0.001 (***, or ^†††^).

## References

[1] Davis FB, Tang HY, Shih A, Keating T, Lansing L, Hercbergs A, Fenstermaker RA, Mousa A, Mousa SA, Davis PJ, Lin HY (2006). Acting via a cell surface receptor, thyroid hormone is a growth factor for glioma cells. Cancer Res.

[2] Lin HY, Tang HY, Shih A, Keating T, Cao G, Davis PJ, Davis FB (2007). Thyroid hormone is a MAPK-dependent growth factor for thyroid cancer cells and is anti-apoptotic. Steroids.

[3] Meng R, Tang HY, Westfall J, London D, Cao JH, Mousa SA, Luidens M, Hercbergs A, Davis FB, Davis PJ, Lin HY (2011). Crosstalk between integrin alphavbeta3 and estrogen receptor-alpha is involved in thyroid hormone-induced proliferation in human lung carcinoma cells. PLoS One.

[4] Chin YT, Yang SH, Chang TC, Changou CA, Lai HY, Fu E, HuangFu WC, Davis PJ, Lin HY, Liu LF (2015). Mechanisms of dihydrotestosterone action on resveratrol-induced anti-proliferation in breast cancer cells with different ERalpha status. Oncotarget.

[5] Tang HY, Lin HY, Zhang S, Davis FB, Davis PJ (2004). Thyroid hormone causes mitogen-activated protein kinase-dependent phosphorylation of the nuclear estrogen receptor. Endocrinology.

[6] Lin HY, Glinsky GV, Mousa SA, Davis PJ (2015). Thyroid hormone and anti-apoptosis in tumor cells. Oncotarget.

[7] Fabian ID, Rosner M, Fabian I, Vishnevskia-Dai V, Zloto O, Shinderman Maman E, Cohen K, Ellis M, Lin HY, Hercbergs A, Davis PJ, Ashur-Fabian O (2015). Low thyroid hormone levels improve survival in murine model for ocular melanoma. Oncotarget.

[8] Cohen K, Flint N, Shalev S, Erez D, Baharal T, Davis PJ, Hercbergs A, Ellis M, Ashur-Fabian O (2014). Thyroid hormone regulates adhesion, migration and matrix metalloproteinase 9 activity via alphavbeta3 integrin in myeloma cells. Oncotarget.

[9] Shinderman-Maman E, Cohen K, Weingarten C, Nabriski D, Twito O, Baraf L, Hercbergs A, Davis PJ, Werner H, Ellis M, Ashur-Fabian O (2015). The thyroid hormone-alphavbeta3 integrin axis in ovarian cancer: regulation of gene transcription and MAPK-dependent proliferation. Oncogene.

[10] Yang SH, Lin HY, Changou CA, Chen CH, Liu YR, Wang J, Jiang X, Luh F, Yen Y (2016). Integrin beta3 and LKB1 are independently involved in the inhibition of proliferation by lovastatin in human intrahepatic cholangiocarcinoma. Oncotarget.

[11] Davis PJ, Davis FB, Lin HY, Mousa SA, Zhou M, Luidens MK (2009). Translational implications of nongenomic actions of thyroid hormone initiated at its integrin receptor. Am J Physiol Endocrinol Metab.

[12] Lin HY, Sun M, Tang HY, Lin C, Luidens MK, Mousa SA, Incerpi S, Drusano GL, Davis FB, Davis PJ (2009). L-Thyroxine vs. 3,5,3′-triiodo-L-thyronine and cell proliferation: activation of mitogen-activated protein kinase and phosphatidylinositol 3-kinase. Am J Physiol Cell Physiol.

[13] Cheng SY, Leonard JL, Davis PJ (2010). Molecular aspects of thyroid hormone actions. Endocr Rev.

[14] Lin HY, Zhang S, West BL, Tang HY, Passaretti T, Davis FB, Davis PJ (2003). Identification of the putative MAP kinase docking site in the thyroid hormone receptor-beta1 DNA-binding domain: functional consequences of mutations at the docking site. Biochemistry.

[15] Rasool M, Naseer MI, Zaigham K, Malik A, Riaz N, Alam R, Manan A, Sheikh IA, Asif M (2014). Comparative study of alterations in tri-iodothyronine (T3) and thyroxine (T4) hormone levels in breast and ovarian cancer. Pak J Med Sci.

[16] Rae MT, Gubbay O, Kostogiannou A, Price D, Critchley HO, Hillier SG (2007). Thyroid hormone signaling in human ovarian surface epithelial cells. J Clin Endocrinol Metab.

[17] Shih A, Lin HY, Davis FB, Davis PJ (2001). Thyroid hormone promotes serine phosphorylation of p53 by mitogen-activated protein kinase. Biochemistry.

[18] Bergh JJ, Lin HY, Lansing L, Mohamed SN, Davis FB, Mousa S, Davis PJ (2005). Integrin alphaVbeta3 contains a cell surface receptor site for thyroid hormone that is linked to activation of mitogen-activated protein kinase and induction of angiogenesis. Endocrinology.

[19] Lin HY, Chin YT, Yang YC, Lai HY, Wang-Peng J, Liu LF, Tang HY, Davis PJ (2016). Thyroid Hormone, Cancer, and Apoptosis. Compr Physiol.

[20] Davis PJ, Mousa SA, Cody V, Tang HY, Lin HY (2013). Small molecule hormone or hormone-like ligands of integrin alphaVbeta3: implications for cancer cell behavior. Horm Cancer.

[21] Cody V, Davis PJ, Davis FB (2007). Molecular modeling of the thyroid hormone interactions with alpha v beta 3 integrin. Steroids.

[22] Lin HY, Su YF, Hsieh MT, Lin S, Meng R, London D, Lin C, Tang HY, Hwang J, Davis FB, Mousa SA, Davis PJ (2013). Nuclear monomeric integrin alphav in cancer cells is a coactivator regulated by thyroid hormone. FASEB J.

[23] Hanna L, Adams M (2006). Prevention of ovarian cancer. Best Pract Res Clin Obstet Gynaecol.

[24] Edson MA, Nagaraja AK, Matzuk MM (2009). The mammalian ovary from genesis to revelation. Endocr Rev.

[25] Beral V, Bull D, Green J, Reeves G (2007). Ovarian cancer and hormone replacement therapy in the Million Women Study. Lancet.

[26] Davis PJ, Sudha T, Lin HY, Mousa SA (2015). Thyroid hormone, hormone analogs, and angiogenesis. Compr Physiol.

[27] Liapis H, Adler LM, Wick MR, Rader JS (1997). Expression of alpha(v)beta3 integrin is less frequent in ovarian epithelial tumors of low malignant potential in contrast to ovarian carcinomas. Hum Pathol.

[28] Landen CN, Kim TJ, Lin YG, Merritt WM, Kamat AA, Han LY, Spannuth WA, Nick AM, Jennnings NB, Kinch MS, Tice D, Sood AK (2008). Tumor-selective response to antibody-mediated targeting of alphavbeta3 integrin in ovarian cancer. Neoplasia.

[29] Boger C, Kalthoff H, Goodman SL, Rocken C (2013). Validation and comparison of anti-alphavbeta3 and anti-alphavbeta5 rabbit monoclonal versus murine monoclonal antibodies in four different tumor entities. Appl Immunohistochem Mol Morphol.

[30] Cannistra SA, Ottensmeier C, Niloff J, Orta B, DiCarlo J (1995). Expression and function of beta 1 and alpha v beta 3 integrins in ovarian cancer. Gynecol Oncol.

[31] Wang Y, Liu J, Lin B, Wang C, Li Q, Liu S, Yan L, Zhang S, Iwamori M (2011). Study on the expression and clinical significances of lewis y antigen and integrin alphav, beta3 in epithelial ovarian tumors. Int J Mol Sci.

[32] Janssen ML, Oyen WJ, Dijkgraaf I, Massuger LF, Frielink C, Edwards DS, Rajopadhye M, Boonstra H, Corstens FH, Boerman OC (2002). Tumor targeting with radiolabeled alpha(v)beta integrin binding peptides in a nude mouse model. Cancer Res.

[33] Zhao Y, Bachelier R, Treilleux I, Pujuguet P, Peyruchaud O, Baron R, Clement-Lacroix P, Clezardin P (2007). Tumor alphavbeta3 integrin is a therapeutic target for breast cancer bone metastases. Cancer Res.

[34] Dijkgraaf I, Kruijtzer JA, Frielink C, Corstens FH, Oyen WJ, Liskamp RM, Boerman OC (2007). Alpha v beta 3 integrin-targeting of intraperitoneally growing tumors with a radiolabeled RGD peptide. Int J Cancer.

[35] Colombo R, Mingozzi M, Belvisi L, Arosio D, Piarulli U, Carenini N, Perego P, Zaffaroni N, De Cesare M, Castiglioni V, Scanziani E, Gennari C (2012). Synthesis and biological evaluation (in vitro and in vivo) of cyclic arginine-glycine-aspartate (RGD) peptidomimetic-paclitaxel conjugates targeting integrin alphaVbeta3. J Med Chem.

[36] Park JW, Zhao L, Willingham M, Cheng SY (2015). Oncogenic mutations of thyroid hormone receptor beta. Oncotarget.

